# Detection of circulating tumor cells and circulating tumor DNA before and after mammographic breast compression in a cohort of breast cancer patients scheduled for neoadjuvant treatment

**DOI:** 10.1007/s10549-019-05326-5

**Published:** 2019-06-24

**Authors:** Daniel Förnvik, Kristina E. Aaltonen, Yilun Chen, Anthony M. George, Christian Brueffer, Robert Rigo, Niklas Loman, Lao H. Saal, Lisa Rydén

**Affiliations:** 1 Department of Translational Medicine, Medical Radiation Physics, Lund University, Malmö, Sweden; 2 Department of Laboratory Medicine, Division of Translational Cancer Research, Lund University, Lund, Sweden; 3 Department of Clinical Sciences Lund, Division of Oncology and Pathology, Lund University, Lund, Sweden; 4 Department of Oncology, Skåne University Hospital, Lund, Sweden; 5 Department of Clinical Sciences Lund, Division of Surgery, Lund University, Lund, Sweden; 6 Department of Surgery and Gastroenterology, Skåne University Hospital, Malmö, Sweden

**Keywords:** Circulating tumor cells, Circulating tumor DNA, Breast compression, Breast cancer, Mammography, Neoadjuvant

## Abstract

**Purpose:**

It is not known if mammographic breast compression of a primary tumor causes shedding of tumor cells into the circulatory system. Little is known about how the detection of circulating biomarkers such as circulating tumor cells (CTCs) or circulating tumor DNA (ctDNA) is affected by breast compression intervention.

**Methods:**

CTCs and ctDNA were analyzed in blood samples collected before and after breast compression in 31 patients with primary breast cancer scheduled for neoadjuvant therapy. All patients had a central venous access to allow administration of intravenous neoadjuvant chemotherapy, which enabled blood collection from superior vena cava, draining the breasts, in addition to sampling from a peripheral vein.

**Results:**

CTC and ctDNA positivity was seen in 26% and 65% of the patients, respectively. There was a significant increase of ctDNA after breast compression in central blood (*p* = 0.01), not observed in peripheral testing. No increase related with breast compression was observed for CTC. ctDNA positivity was associated with older age (*p* = 0.05), and ctDNA increase after breast compression was associated with high Ki67 proliferating tumors (*p* = 0.04). CTCs were more abundant in central compared to peripheral blood samples (*p* = 0.04).

**Conclusions:**

There was no significant release of CTCs after mammographic breast compression but more CTCs were present in central compared to peripheral blood. No significant difference between central and peripheral levels of ctDNA was observed. The small average increase in ctDNA after breast compression is unlikely to be clinically relevant. The results give support for mammography as a safe procedure from the point of view of CTC and ctDNA shedding to the blood circulation. The results may have implications for the standardization of sampling procedures for circulating tumor markers.

**Electronic supplementary material:**

The online version of this article (10.1007/s10549-019-05326-5) contains supplementary material, which is available to authorized users.

## Introduction

Circulating tumor markers such as circulating tumor cells (CTCs) and circulating tumor DNA (ctDNA) can be found in the blood of cancer patients. As a liquid biopsy, these markers complement solid biopsies and have the advantage of being physically more accessible and patient-friendly than traditional tissue biopsies. This provides a possibility for prognosis prediction, closer monitoring of treatment response and disease progression, identification of drug targets, as well as an opportunity for early detection of recurrence. The presence of CTCs in the blood of patients with primary breast cancer has been shown to be an independent predictor of decreased disease-free and overall survival [1, 2], but the treatment predictive value of the cells is still under debate [3, 4]. The CTC methodology in primary breast cancer is also limited by the low number of detected cells, which makes enumeration and evaluation statistically challenging [5]. ctDNA, the cell-free DNA that originates from cancer cells, is a promising biomarker whose prognostic and treatment predictive power is emerging [6, 7]. Recent studies have shown that quantification of specific mutations in ctDNA can be associated with early detection of metastases and therapy resistance in breast cancer as well as in other diagnoses [8–12].

The risk that tumor cells are released into the bloodstream from a primary tumor during surgical interventions has been addressed in a few studies [13–17], although how and when tumor cells are shed as well as the clinical importance of this release is poorly understood [18]. Animal studies have also found that physical manipulation of a primary tumor by applying pressure to it causes tumor cell dissemination [19–21]. We have previously investigated if mammographic breast compression in patients with an already present breast tumor could cause shedding of tumor cells to the peripheral circulation [22]. We found no indications that this would be the case in a pilot study of 24 patients with primary breast cancer.

However, the configuration of the human blood circulation can cause tumor cells released from the breast to pass through the capillary vasculature of the lungs before reaching the peripheral blood vessels. In our previous study [22], CTCs captured only in the peripheral blood might have resulted in an underestimation of CTC number. It has been shown that a higher number of CTCs can be found in central compared to peripheral venous blood in patients with metastatic breast cancer [23] as well as in other diagnoses [24–26]. Animal studies of colon carcinoma cells have shown that the majority (80–100%) of tumor cells could be trapped in the capillary bed of the first organ they encounter [27]. In breast cancer, an autopsy study by Peeters et al. [28] showed that CTCs were trapped in the lung microvasculature in four of the nine patients who all had high CTC counts (> 100). Thus, it is likely that the number of CTCs found in the peripheral blood system is not representative of a possible release of tumor cells from the primary tumor during manipulation such as breast compression during mammography or surgery. The difference in CTC number between central and peripheral blood is possibly even more pronounced after specific interventions compared to a more steady-state-like condition of metastatic disease [24, 26].

To our knowledge, ctDNA levels have not been used to study a possible release of tumor cells or tumor cell debris after breast compression or any other mechanical intervention in breast cancer. Relatively few studies have so far compared the levels of both CTCs and ctDNA in the same clinical patient cohort at identical time points and our understanding of the relationship between the two liquid tumor markers is limited. However, both the level of ctDNA and the number of CTCs have been shown to have a prognostic value in mainly metastatic breast cancer cohorts [29, 30]. Mutation analysis of ctDNA and single CTCs suggests that ctDNA reflects the heterogeneity of mutations found in individual CTCs [30], but ctDNA levels have been found to have a higher correlation with tumor burden than CTCs [29].

The aim of this study was to investigate how the presence of CTCs and ctDNA are affected by breast compression during mammography in patients with primary breast cancer. Special emphasis was made on comparing circulating tumor marker burden between the central and peripheral blood circulation.

## Materials and methods

### Patient cohort and clinical parameters

The patient cohort comprises preoperative patients within the ongoing SCAN-B trial (Clinical Trials ID NCT02306096) at Lund University and Skåne University Hospital, Sweden [31, 32]. During 2015–2016, 31 patients scheduled for neoadjuvant therapy volunteered to do an extra mammography after diagnosis and were included in the present study. The patient mean age was 51.9 years (range 33–74 years) and the mean compressed breast thickness and applied compression force during the examination were 55.8 mm (range 26.5–77.0 mm) and 103.4 N (range 71.5–123.1 N), respectively, as indicated by the mammography system (Mammomat Inspiration, Siemens Healthineers, Erlangen, Germany). All patients gave written informed consent and the study was approved by the Regional Ethical Review Board in Lund, Sweden (diary number 2014/521).

Clinical data including biomarker expression, histological subtype, and nodal status were retrieved from pathology reports and the patient’s clinical charts. Information on biomarker expression was based on analysis from the core needle biopsy before initiation of neoadjuvant therapy. Estrogen receptor (ER) positivity was defined as ≥ 10% positive cancer cells, human epidermal growth factor receptor 2 (HER2) positivity was defined by immunohistochemistry (IHC) or in situ hybridization (ISH) as (IHC3+) or ISH-positive cells, and Ki67 positivity was defined as > 20% positive cancer cells. Information from mammograms, ultrasound images, and breast tomosynthesis was compiled into one measure of tumor size.

### Blood sampling

All patients had a central venous access to allow administration of intravenous neoadjuvant chemotherapy, which enabled blood collection from superior vena cava, draining the breasts. A dedicated research nurse attended the patient during the mammography examination and acquired blood samples before and after mammographic breast compression, first from central venous access and secondly from a peripheral vein at both occasions. The median time for blood sampling after compression was 2 min (range 0–5 min) for central blood samples and 7 min (range 5–23) for peripheral blood samples. At each time point, 10 ml whole blood was collected in CellSave tubes (Menarini Silicon Biosystems, Bologna, Italy) for CTC analysis and 10 ml whole blood was collected in Cell-Free DNA Blood Collection Tubes (Streck Inc., Omaha, USA) for ctDNA analysis. The blood samples were transported at room temperature and subsequent analyses were performed within 96 h after sample taking.

### CTC analysis

The blood samples were analyzed for CTC number using the FDA-approved CellSearch^©^ system (Menarini Silicon Biosystems). Briefly, a ferrofluid-conjugated epithelial cell adhesion molecule (EpCAM)-directed antibody was used to separate CTCs from the majority of white blood cells. Fluorescent staining with DAPI (nuclear staining), cytokeratin (CK) 8, 18, 19-directed PE-conjugated antibodies, and CD45-directed APC-conjugated antibodies were applied to identify CTCs (DAPI +/CK+/CD45–). Two independent and accredited technicians manually evaluated images of CK + events selected automatically by the CellTracks II system (Menarini Silicon Biosystems). The method has been described in detail elsewhere [33]. Cut-off for CTC positivity was ≥ 1 CTC/7.5 ml blood as suggested by a recent review of primary breast cancer [1].

### ctDNA analysis

Candidate somatic mutations for ctDNA measurement were obtained from RNA sequencing (RNA-seq) data generated within SCAN-B [31, 34]. Twenty-eight of the 31 patients had available tumor RNA-seq data. Sequencing, base calling, FASTQ file processing, and filtering were performed as previously described [34]. Using a Snakemake workflow, reads in FASTQ format were aligned to the human reference genome GRCh38.p8 (including alternative sequences and decoys, and patched with dbSNP Build 147) using HISAT2 2.0.5 [35] (with default options except --rna-strandness RF, --rg-id ${ID_NAME}, --rg PL:illumina, --rg PU:${UNIT}, --rg SM:${SAMPLE}), and duplicate reads were marked using SAMBLASTER 0.1.24. Variants were called using VarDict-Java 1.5.0 [36] (with default options except -f 0.02, -N ${SAMPLE}, -b ${BAM_FILE}, -c 1, -S 2, -E 3, -g 4, -Q 10, -r 2, -q 20), and annotated with dbSNP build 150 and COSMIC v84 using vcfanno 0.2.8 [37].

From the RNA-seq mutation calling, one somatic mutation for each patient was selected for IBSAFE assay design for ultrasensitive mutation detection. IBSAFE^©^ (SAGA Diagnostics AB, Lund, Sweden) is an enhanced droplet digital PCR technology with significantly improved sensitivity and specificity, allowing for quantification of alleles to 0.001% mutant allele frequency (MAF) [George et al. manuscript in preparation]. IBSAFE assays targeting a somatic mutation were designed for 20 patients and the assays validated using 6 ng of corresponding tumor DNA as positive control and 180 ng of human normal genomic DNA (Promega, Madison, USA) as negative control, confirming a lower limit of detection of at least 0.0017% MAF for each assay.

Whole blood collected in Streck tubes were centrifuged at 2000×*g* for 15 min at room temperature to fractionate plasma, followed by clearing of the plasma fraction by centrifugation at 10,000×*g* for 15 min at 4 °C. Cell-free DNA was isolated using the QIAamp Circulating Nucleic Acid Kit or the QIAamp MinElute ccfDNA Midi Kit (Qiagen, Hilden, Germany), of which 20% of the eluate used for IBSAFE reactions and measurement of mutant and wild-type ctDNA copies and calculation of MAF.

### Statistical analysis

Clinical and patient-specific characteristics were compared between patients that had ≥ 1 CTC/≥ 0.01% MAF present in any sample and patients with 0 CTCs/0% MAF in all samples. Agreement between CTC- and ctDNA-positive patients was analyzed using Cohen’s kappa statistics. The Mann–Whitney U-test was used to compare the distribution of continuous variables. For categorical variables, Fisher’s exact test was used in all comparisons due to less than five expected cases in at least one of the groups in all cross-tables. Statistical analysis of all characteristics was also performed between patients that had an increase in CTC number/% MAF after compression with patients that did not have an increase in CTC number/% MAF after compression. A non-parametric Wilcoxon signed-rank test was applied to test for CTC differences/% MAF changes between before and after compression. For comparison between central and peripheral CTC/ctDNA measurements, a sign test was used.

All statistical analyses were performed in IBM SPSS Statistics (version 24, IBM, Armonk, NY, USA) and *p* values < 0.05 were considered significant.

## Results

**Fig. 1:**
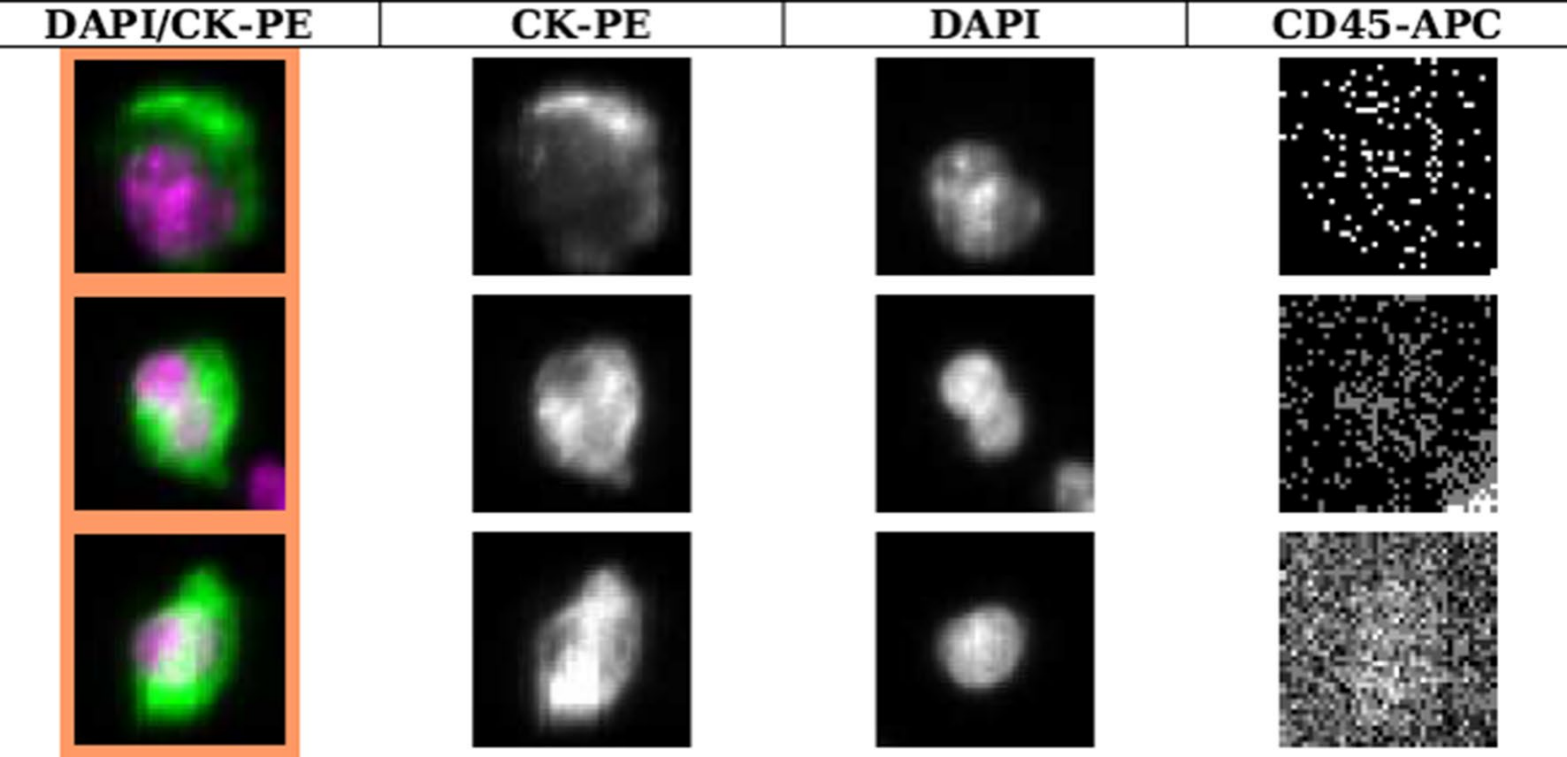
Examples of CTCs detected with the CellSearch system from a patient in the study

In total, 8/31 patients (26%) had ≥ 1 CTC in at least one of the blood samples taken before or after mammographic breast compression (Fig. 1). Correspondingly, 13/20 patients (65%) had ≥ 0.01% MAF and were defined as ctDNA positive. No agreement was found between CTC- and ctDNA-positive patients (κ = 0.02, *p *= 0.92) (A plot of CTC count versus % MAF can be found in supplementary Fig. S1).

Patient and tumor characteristics of the whole cohort, as well as of CTC/ctDNA-positive and CTC/ctDNA-negative patients separately, are shown in Table 1. No patient or pathologic characteristics were statistically associated with CTC positivity. Larger tumor size, non-ductal histological subtype, and older age were more predominant in the CTC-positive group but the difference was not statistically significant. ctDNA positivity was associated with higher age (*p *= 0.05). Higher Ki67, ductal histological type, and triple-negative breast cancer were more predominant in ctDNA-positive patients, without reaching statistical significance (Table 1). Notably, 4/4 triple-negative breast cancers (TNBC) and 4/4 T4 staged cancers were all ctDNA positive.

**Table 1:** Comparison of patient and tumor characteristics between patients positive for CTCs and ctDNA (≥ 1 CTC/≥ 0.01% MAF) and patients with no CTCs/% MAF

	Total (*N *= 31)	CTC negative (*N *= 23)	CTC positive (*N *= 8)	*p* value	Total (*N *= 20)	ctDNA negative (*N *= 7)	ctDNA positive (*N *= 13)	*p* value
Age (years)								
Median (range)	50 (33–74)	47 (33–74)	56 (43–71)	0.21^a^	51 (35–74)	46 (35–62)	58 (40–74)	0.05^a^
< 50	16	13	3	0.43^b^	10	5	5	0.35^b^
≥ 50	15	10	5		10	2	8	
Tumor size and stage								
Median size, mm (range)	30 (4–90)	30 (4–90)	38 (16–80)	0.21^a^	30 (4–80)	24 (8–80)	30 (4–80)	0.60^a^
T1 (< 20 mm)	8	7	1	0.64^b^	6	3	3	0.61^b^
T2–T4 (20 mm or higher)	23	16	7		14	4	10	
Nodal stage								
N0	4	3	1	1.0^b^	2	0	2	0.52^b^
N+	27	20	7		18	7	11	
ER								
Negative (10% or lower)	8	6	2	1.0^b^	4	0	4	0.25^b^
Positive (> 10%)	23	17	6		16	7	9	
HER2								
Negative	25	18	7	1.0^b^	17	5	12	0.27^b^
Positive	6	5	1		3	2	1	
Ki67								
Median % of cells stained (range)	45 (15–95)	45 (20–95)	49 (15–90)	0.61^a^	40 (15–90)	30 (15–90)	45 (20–90)	0.19^a^
Low (20% or lower)	3	2	1	1.0^b^	3	1	2	1.0^b^
High (> 20%)	28	21	7		17	6	11	
Breast cancer subtype								
ER+	18	12	6	0.63^b^	13	5	8	0.20^b^
HER2+	6	5	1		3	2	1	
TNBC	7	6	1		4	0	4	
Multifocality								
No	22	17	5	0.64^b^	15	4	11	0.29^b^
Yes	8	5	3		5	3	2	
Missing	1	1						
Histological subtype								
Ductal	23	19	4	0.15^b^	13	3	10	0.17^b^
Other	8	4	4		7	4	3	
Detection mode								
Screening	9	7	2	1.0^b^	8	2	6	0.64^b^
Symptomatic	22	16	6		12	5	7	

^a^Mann–Whitney *U*-test

^b^Fisher’s exact test

**Fig. 2:**
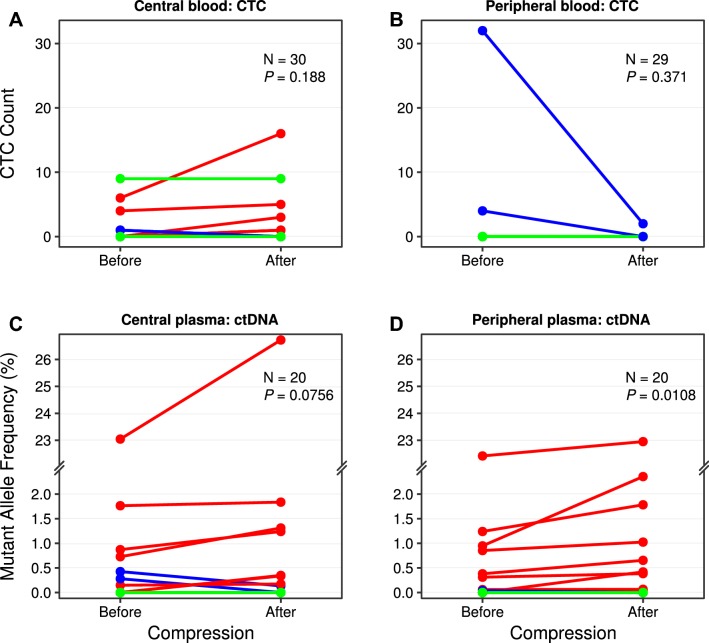
The number of CTCs found before and after mammographic breast compression in central venous access (**a**), where two patients are represented by a line going from 0 to 1 CTC and from 1 to 0 CTCs, respectively. The corresponding number of CTCs before and after compression in peripheral blood (**b**). Figures for mutant allele frequency before and after breast compression in central (**c**), where two patients are represented by a line from 0 to approximately 0.35, and peripheral (**d**) plasma, where two patients are represented by a line going from 0 to approximately 0.04 and from approximately 0.03 to 0, respectively. Patients with an increasing value are plotted with red lines, decreasing values in blue, and constant values in green. All patients that did not have any circulating tumor markers are summarized in one line at number/frequency = 0

Thirty patients had CTC results from the central blood sample before and after breast compression and 22 of these patients had 0 CTCs at both time points. Five of eight patients with detectable CTCs had an increased number of CTCs after compression (*p *= 0.19) (Fig. 2a). The average CTC increase was 3.2 cells (median 1.0 cell). Only two evaluable patients had detectable CTCs in the peripheral blood sample (Fig. 2b). Both central and peripheral % MAF generally increased after compression with the latter reaching significance (*p *= 0.08 and *p *= 0.01) (Fig. 2c, d). The average increase of % MAF was relatively small, 0.77 and 0.35 (median 0.35 and 0.22% MAF) for central and peripheral, respectively. Of the 20 patients with assessable ctDNA samples before and after breast compression, eleven and eight patients had 0% MAF in central and peripheral plasma samples, respectively, at both time points.

The median fraction of Ki67-positive cells was 66% (range 30–90%) in the five patients that had an increase in CTC number after compression, compared to 45% (range 15–95%) in patients with no increase (*p *= 0.31) (Supplementary Table 1). Also, Ki67 fraction was significantly higher in the group with increasing ctDNA after compression, 45% versus 30% (*p *= 0.04) (Supplementary Table 2). No other factors were differentially expressed between patients with an increase in CTCs/ctDNA levels and patients with a stable or a decrease in CTCs/ctDNA levels after compression. However, the histological type of the primary tumor seemed to differ between patients with an increase in the number of CTCs and patients with an increase in the levels of ctDNA after compression (Supplementary Tables 1 and 2).

**Fig. 3:**
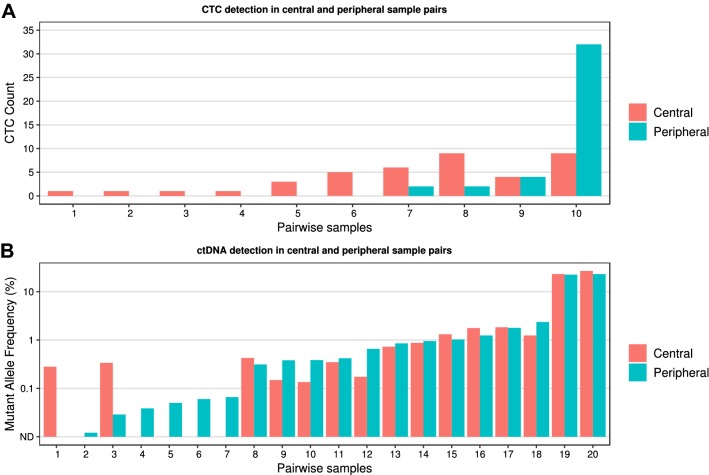
CTC (**a**) and ctDNA (**b**) detection in central and peripheral sample pairs. In 8/10 CTC-positive pairwise samples, a higher number of CTCs was detected in the central compared to the peripheral blood sample (*p* = 0.04). In pairwise samples 1–6, no CTCs were found in the peripheral blood sample. In 12/20 ctDNA pairwise samples, a higher mutant allele fraction was found in the peripheral plasma sample (*p* = 0.50). In pairwise samples 2, 4–7, no ctDNA was detected centrally, and in sample 1, no ctDNA was detected peripherally

CTCs were more abundant in central compared to peripheral blood in 8/10 positive samples (*p *= 0.04) (Fig. 3a). Forty-nine comparisons between central and peripheral blood contained 0 CTCs in both samples. There was no significant difference in % MAF levels between central and peripheral sampling (8/20 favoring higher % MAF in the central blood sample, *p *= 0.50) (Fig. 3b). Twenty comparisons between central and peripheral blood contained 0% MAF in both samples.

## Discussion

CTCs were detected in 26% of the patients before start of neoadjuvant therapy for primary breast cancer. This is in line with a recent meta-analysis where 25.2% of breast cancer patients had CTCs before onset of neoadjuvant chemotherapy (deemed independent of blood sampling volume) [2]. ctDNA was positive in 65% of the patients. The concordance between CTCs and ctDNA has been shown to be higher in metastatic breast cancer patients [29] as compared to what was found in this study, which is most likely due to the lower rate of CTCs in primary breast cancer (Supplementary Fig. S1). ctDNA positivity, defined as ≥ 0.01% MAF in this study, was associated with higher age (*p *= 0.05) and a trend was noted that a more aggressive tumor phenotype, including high Ki67, TNBC, and T4 staged cancers, favors ctDNA positivity (not statistically significant).

For CTCs, no increase was seen in either central or peripheral blood after mammographic breast compression (*p *= 0.19 and *p *= 0.37, respectively). However, both central and peripheral ctDNA levels increased after breast compression (*p *= 0.08 and *p *= 0.01, respectively). Only one patient had a CTC count difference of > 5 cells/7.5 ml between samples taken before and after compression (Fig. 2a). This suggests a lack of a larger bolus release of cells during breast compression of women with primary breast cancer. The central blood samples were drawn on average 2 min after compression and, according to an animal study, the release of malignant cells starts at the manipulation procedure and stays elevated up to 60 min [19]. To the best of the authors’ knowledge, no published study has investigated how ctDNA levels vary with manipulation of a primary tumor with regard to applied pressure. The increase of ctDNA after breast compression found in this study can be considered relatively small, with only one case going from % MAF 0.95 to 2.36 which possibly could affect prognostication (Fig. 2d) [8]. High Ki67 were associated with increased ctDNA levels (*p *= 0.04) (Supplementary Table 2).

As hypothesized, CTCs were in general significantly more likely to be present in central than in peripheral blood samples (*p *= 0.04). Six patients presented CTCs only in the central samples (Fig. 3a) suggesting a differential CTC yield depending on sample location. The results are comparable to the work by Peeters et al. [23] in metastatic breast cancer but no data from studies involving differential blood sampling of primary breast cancer are hitherto available. This differential yield was not seen for ctDNA (*p *= 0.50). Since ctDNA is a much smaller moiety and soluble in the blood, we speculate that ctDNA is much less affected by physical hindrance in the capillaries as compared to CTCs. Hence, ctDNA blood sampling is independent of blood drawing location, a finding that could contribute to the definition of clinical sampling routines in primary breast cancer for ctDNA.

The major limitation of this study was the small sample size and low count of CTCs, despite that a total of up to 40 ml whole blood was drawn from each patient. The statistical nature of CTC sampling has been described by Tibbe et al. [5]. Due to the low sample size, a possible difference between CTCs detection before and after breast compression may have been underestimated. Similarly, the ctDNA analysis was limited by a relatively low plasma input volume, and therefore a limited number of genome equivalents being analyzed for the presence of mutations.

When CTCs and ctDNA markers are implemented into clinical routine, our understanding of how the concentrations fluctuate during different interventions should be better understood. The women in this cohort are continuously being monitored and follow-up data will be available and presented in future publications.

## Conclusion

In summary, there was no significant release of CTCs after mammographic breast compression but more CTCs were present in central compared to peripheral blood. There was a small average increase in ctDNA levels after breast compression, unlikely to be clinically relevant, and no difference between central and peripheral levels was found.

## Electronic supplementary material

Below is the link to the electronic supplementary material.

Supplementary material 1 (EPS 42 kb)

Supplementary material 2 (DOC 63 kb)

Supplementary material 3 (DOC 61 kb)

## Abbreviations

CTC: Circulating tumor cells
ctDNA: Circulating tumor DNA
RNA-seq: RNA sequencing
MAF: Mutant allele frequency
TNBC: Triple-negative cancers

## References

[1] Janni WJ, Rack B, Terstappen LW (2016). Pooled analysis of the prognostic relevance of circulating tumor cells in primary breast cancer. Clin Cancer Res.

[2] Bidard FC, Michiels S, Riethdorf S (2018). Circulating tumor cells in breast cancer patients treated by neoadjuvant chemotherapy: a meta-analysis. J Natl Cancer Inst.

[3] Bardelli A, Pantel K (2017). Liquid biopsies, what we do not know (yet). Cancer Cell.

[4] Yan WT, Cui X, Chen Q (2017). Circulating tumor cell status monitors the treatment responses in breast cancer patients: a meta-analysis. Sci Rep.

[5] Tibbe AG, Miller MC, Terstappen LW (2007). Statistical considerations for enumeration of circulating tumor cells. Cytometry A.

[6] Wan JC, Massie C, Garcia-Corbacho J (2017). Liquid biopsies come of age: towards implementation of circulating tumour DNA. Nat Rev Cancer.

[7] Beddowes E, Sammut SJ, Gao M, Caldas C (2017). Predicting treatment resistance and relapse through circulating DNA. Breast.

[8] Olsson E, Winter C, George A (2015). Serial monitoring of circulating tumor DNA in patients with primary breast cancer for detection of occult metastatic disease. EMBO Mol Med.

[9] Murtaza M, Dawson SJ, Tsui DW (2013). Non-invasive analysis of acquired resistance to cancer therapy by sequencing of plasma DNA. Nature.

[10] Spindler KL, Pallisgaard N, Andersen RF, Jakobsen A (2014). Changes in mutational status during third-line treatment for metastatic colorectal cancer–results of consecutive measurement of cell free DNA, KRAS and BRAF in the plasma. Int J Cancer.

[11] Scholer LV, Reinert T, Orntoft MW (2017). Clinical implications of monitoring circulating tumor DNA in patients with colorectal cancer. Clin Cancer Res.

[12] Loman N, Saal LH (2016). The state of the art in prediction of breast cancer relapse using cell-free circulating tumor DNA liquid biopsies. Ann Transl Med.

[13] Sandri MT, Zorzino L, Cassatella MC (2010). Changes in circulating tumor cell detection in patients with localized breast cancer before and after surgery. Ann Surg Oncol.

[14] Papavasiliou P, Fisher T, Kuhn J (2010). Circulating tumor cells in patients undergoing surgery for hepatic metastases from colorectal cancer. Proc (Bayl Univ Med Cent).

[15] Koch M, Kienle P, Hinz U (2005). Detection of hematogenous tumor cell dissemination predicts tumor relapse in patients undergoing surgical resection of colorectal liver metastases. Ann Surg.

[16] van Dalum G, van der Stam GJ, Tibbe AG (2015). Circulating tumor cells before and during follow-up after breast cancer surgery. Int J Oncol.

[17] Hashimoto M, Tanaka F, Yoneda K (2014). Significant increase in circulating tumour cells in pulmonary venous blood during surgical manipulation in patients with primary lung cancer. Interact Cardiovasc Thorac Surg.

[18] Martin OA, Anderson RL, Narayan K, MacManus MP (2017). Does the mobilization of circulating tumour cells during cancer therapy cause metastasis?. Nat Rev Clin Oncol.

[19] Juratli MA, Sarimollaoglu M, Siegel ER (2014). Real-time monitoring of circulating tumor cell release during tumor manipulation using in vivo photoacoustic and fluorescent flow cytometry. Head Neck.

[20] Juratli MA, Siegel ER, Nedosekin DA (2015). In vivo long-term monitoring of circulating tumor cells fluctuation during medical interventions. PLoS ONE.

[21] Nishizaki T, Matsumata T, Kanematsu T (1990). Surgical manipulation of VX2 carcinoma in the rabbit liver evokes enhancement of metastasis. J Surg Res.

[22] Förnvik D, Andersson I, Dustler M (2013). No evidence for shedding of circulating tumor cells to the peripheral venous blood as a result of mammographic breast compression. Breast Cancer Res Treat.

[23] Peeters DJE, Van den Eynden GG, van Dam PJ (2011). Circulating tumour cells in the central and the peripheral venous compartment in patients with metastatic breast cancer. Br J Cancer.

[24] Jiao LR, Apostolopoulos C, Jacob J (2009). Unique localization of circulating tumor cells in patients with hepatic metastases. J Clin Oncol.

[25] Deneve E, Riethdorf S, Ramos J (2013). Capture of viable circulating tumor cells in the liver of colorectal cancer patients. Clin Chem.

[26] Reddy RM, Murlidhar V, Zhao L (2016). Pulmonary venous blood sampling significantly increases the yield of circulating tumor cells in early-stage lung cancer. J Thorac Cardiovasc Surg.

[27] Mizuno N, Kato Y, Izumi Y (1998). Importance of hepatic first-pass removal in metastasis of colon carcinoma cells. J Hepatol.

[28] Peeters DJ, Brouwer A, Van den Eynden GG (2015). Circulating tumour cells and lung microvascular tumour cell retention in patients with metastatic breast and cervical cancer. Cancer Lett.

[29] Dawson SJ, Tsui DW, Murtaza M (2013). Analysis of circulating tumor DNA to monitor metastatic breast cancer. N Engl J Med.

[30] Shaw JA, Guttery DS, Hills A (2016). Mutation analysis of cell-free DNA and single circulating tumor cells in metastatic breast cancer patients with high CTC counts. Clin Cancer Res.

[31] Saal LH, Vallon-Christersson J, Hakkinen J (2015). The Sweden cancerome analysis network-breast (SCAN-B) initiative: a large-scale multicenter infrastructure towards implementation of breast cancer genomic analyses in the clinical routine. Genome Med.

[32] Rydén L, Loman N, Larsson C (2018). Minimizing inequality in access to precision medicine in breast cancer by real-time population-based molecular analysis in the SCAN-B initiative. Br J Surg.

[33] Cristofanilli M, Budd GT, Ellis MJ (2004). Circulating tumor cells, disease progression, and survival in metastatic breast cancer. N Engl J Med.

[34] Brueffer C, Vallon-Christersson J, Grabau D (2018). Clinical value of rna sequencing-based classifiers for prediction of the five conventional breast cancer biomarkers: a report from the population-based multicenter Sweden cancerome analysis network—breast initiative. JCO Precis Oncol.

[35] Kim D, Langmead B, Salzberg SL (2015). HISAT: a fast spliced aligner with low memory requirements. Nat Methods.

[36] Lai Z, Markovets A, Ahdesmaki M (2016). VarDict: a novel and versatile variant caller for next-generation sequencing in cancer research. Nucleic Acids Res.

[37] Pedersen BS, Layer RM, Quinlan AR (2016). Vcfanno: fast, flexible annotation of genetic variants. Genome Biol.

