# Supplementary material for: Detection of circulating tumor cells and circulating tumor DNA before and after mammographic breast compression in a cohort of breast cancer patients scheduled for neoadjuvant treatment
Source: Breast Cancer Res Treat. 2019 Jun 24;177(2):447–55. doi: 10.1007/s10549-019-05326-5 (PMC6661025; doi:10.1007/s10549-019-05326-5)
Supplement: Supplementary file 2 — Supplementary material 2 (DOC 63 kb) [file 10549_2019_5326_MOESM2_ESM.doc]

**Supplementary Table 1.** Comparison between patients with an increase in CTC number after breast compression and patients with no increase.

|  | Total (*N*=31) | No change or decrease in CTC-number (*N*=26) | Increase in CTC number (*N*=5) | *P*-value |
| --- | --- | --- | --- | --- |
| **Age (years)** |  |  |  |  |
| Median (range) | 50 (33-74) | 49 (33-74) | 53 (43-71) | 0.56^a^ |
| <50 | 16 | 14 | 2 | 0.65^b^ |
| ≥50 | 15 | 12 | 3 |  |
| **Tumor size and stage** |  |  |  |  |
| Median size, mm (range) | 30 (4-90) | 31 (4-90) | 36 (16-80) | 0.91^a^ |
| T1 (<20 mm) | 8 | 7 | 1 | 1.0^b^ |
| T2-T4 (20mm or higher) | 23 | 19 | 4 |  |
| **Nodal stage** |  |  |  |  |
| N0 | 4 | 3 | 1 | 0.53^b^ |
| N+ | 27 | 23 | 4 |  |
| **ER** |  |  |  |  |
| Negative (10% or lower) | 8 | 6 | 2 | 0.58^b^ |
| Positive (>10%) | 23 | 20 | 3 |  |
| **HER2** |  |  |  |  |
| Negative | 25 | 21 | 4 | 1.0^b^ |
| Positive | 6 | 5 | 1 |  |
| **Ki67** |  |  |  |  |
| Median % of cells stained (range) | 45 (15-95) | 45 (15-95) | 66 (30-90) | 0.31^a^ |
| Low (20% or lower) | 3 | 3 | 0 | 1.0^b^ |
| High (>20%) | 28 | 23 | 5 |  |
| **Breast cancer subtype** |  |  |  |  |
| ER+ | 18 | 15 | 3 | 1.0^b^ |
| HER2+ | 6 | 5 | 1 |  |
| TNBC | 7 | 6 | 1 |  |
| **Multifocality** |  |  |  |  |
| No | 22 | 18 | 4 | 1.0^b^ |
| Yes | 8 | 7 | 1 |  |
| Missing | 1 | 1 |  |  |
| **Histological subtype** |  |  |  |  |
| Ductal | 23 | 20 | 3 | 0.58^b^ |
| Other | 8 | 6 | 2 |  |
| **Detection mode** |  |  |  |  |
| Screening | 9 | 8 | 1 | 1.0^b^ |
| Symptomatic | 22 | 18 | 4 |  |

^a^Mann-Whitney U-test

^b^Fisher’s exact test
