# Supplementary material for: Detection of circulating tumor cells and circulating tumor DNA before and after mammographic breast compression in a cohort of breast cancer patients scheduled for neoadjuvant treatment
Source: Breast Cancer Res Treat. 2019 Jun 24;177(2):447–55. doi: 10.1007/s10549-019-05326-5 (PMC6661025; doi:10.1007/s10549-019-05326-5)
Supplement: Supplementary file 3 — Supplementary material 3 (DOC 61 kb) [file 10549_2019_5326_MOESM3_ESM.doc]

**Supplementary Table 2.** Comparison between patients with an increase in % MAF level after breast compression and patients with no increase.

|  | Total (*N*=20) | No change or decrease in % MAF (*N*=11) | Increase in % MAF (*N*=9) | *P*-value |
| --- | --- | --- | --- | --- |
| **Age (years)** |  |  |  |  |
| Median (range) | 51 (35-74) | 52 (35-68) | 50 (40-74) | 0.71^a^ |
| <50 | 10 | 5 | 5 | 1.0^b^ |
| ≥50 | 10 | 6 | 4 |  |
| **Tumor size and stage** |  |  |  |  |
| Median size, mm (range) | 30 (4-80) | 24 (4-80) | 30 (19-70) | 0.62^a^ |
| T1 (<20 mm) | 6 | 5 | 1 | 0.16^b^ |
| T2-T4 (20mm or higher) | 14 | 6 | 8 |  |
| **Nodal stage** |  |  |  |  |
| N0 | 2 | 1 | 1 | 1.0^b^ |
| N+ | 18 | 10 | 8 |  |
| **ER** |  |  |  |  |
| Negative (10% or lower) | 4 | 1 | 3 | 0.29^b^ |
| Positive (>10%) | 16 | 10 | 6 |  |
| **HER2** |  |  |  |  |
| Negative | 17 | 9 | 8 | 1.0^b^ |
| Positive | 3 | 2 | 1 |  |
| **Ki67** |  |  |  |  |
| Median % of cells stained (range) | 40 (15-90) | 30 (15-90) | 50 (20-90) | 0.04^a^ |
| Low (20% or lower) | 3 | 2 | 1 | 1.0^b^ |
| High (>20%) | 17 | 9 | 8 |  |
| **Breast cancer subtype** |  |  |  |  |
| ER+ | 13 | 8 | 5 | 0.43^b^ |
| HER2+ | 3 | 2 | 1 |  |
| TNBC | 4 | 1 | 3 |  |
| **Multifocality** |  |  |  |  |
| No | 15 | 8 | 7 | 1.0^b^ |
| Yes | 5 | 3 | 2 |  |
| **Histological subtype** |  |  |  |  |
| Ductal | 13 | 5 | 8 | 0.07^b^ |
| Other | 7 | 6 | 1 |  |
| **Detection mode** |  |  |  |  |
| Screening | 8 | 5 | 3 | 0.67^b^ |
| Symptomatic | 12 | 6 | 6 |  |

^a^Mann-Whitney U-test

^b^Fisher’s exact test
